# Replicative and radiation-induced aging: a comparison of gene expression profiles

**DOI:** 10.18632/aging.101921

**Published:** 2019-04-19

**Authors:** Alexander M. Aliper, Marine E. Bozdaganyan, Philipp S. Orekhov, Alex Zhavoronkov, Andreyan N. Osipov

**Affiliations:** 1 Inсilico Medicine, Inc., Baltimore, MD 21218, USA; 2 State Research Center-Burnasyan Federal Medical Biophysical Center of Federal Medical Biological Agency (SRC-FMBC), Moscow 123098, Russia; 3 Moscow Institute of Physics and Technology, Dolgoprudny, Russia

**Keywords:** replicative aging, ionizing radiation, transcriptome analysis, signal pathway transduction

## Abstract

All living organisms are subject to the aging process and experience the effect of ionizing radiation throughout their life. There have been a number of studies that linked ionizing radiation process to accelerated aging, but comprehensive signalome analysis of both processes was rarely conducted. Here we present a comparative signaling pathway based analysis of the transcriptomes of fibroblasts irradiated with different doses of ionizing radiation, replicatively aged fibroblasts and fibroblasts collected from young, middle age and old patients. We demonstrate a significant concordance between irradiation-induced and replicative senescence signalome signatures of fibroblasts. Additionally, significant differences in transcriptional response were also observed between fibroblasts irradiated with high and low dose. Our data shows that the transcriptome of replicatively aged fibroblasts is more similar to the transcriptome of the cells irradiated with 2 Gy, than with 5 сGy.This work revealed a number of signaling pathways that are shared between senescence and irradiation processes and can potentially be targeted by the new generation of gero- and radioprotectors.

## Introduction

All living organisms are constantly exposed to ionizing radiation coming from radionuclides of natural origin, cosmic rays and multiple anthropogenic sources, such as weapon tests, nuclear reprocessing, and nuclear accidents. In biological systems, ionizing radiation leads to ionization of molecules and direct DNA damage, formation of free radicals, and ultimately to the generation of reactive oxygen species (ROS) and reactive nitrogen species (RNS). Increased cellular concentrations of ROS/RNS result in oxidative stress and secondary indirect DNA damage [1]. It is believed that exposure of cells to low doses of radiation can activate cellular defense systems, while high doses result in significant damage to DNA and other macromolecules, cell death and cancer [2].

The effects of ionizing radiation (IR) are to some extent analogous to the processes observed in hereditary progeroid syndromes, and similar to premature aging. Segmental progerias (dyskeratosis congenita, Werner syndrome, Bloom syndrome and ataxia telangiectasia (AT)) have only some symptoms of “accelerated aging”, mostly they are characterized by impaired DNA repair and genetic instability. Hofer et al. [3] hypothesized that only some progeroid syndromes with symptoms of alopecia (hair loss), osteoporosis, and nail atrophy are associated with telomere shortening, whereas in Bloom syndrome, for example, telomere shortening is not observed. AT syndrome is caused by a defect in the ATM (Ataxia-Telangiectasia Mutated) protein, whose normal function is to ensure DNA repair during cell division. If DNA damage is beyond repair, ATM becomes a mediator of programmed cell death (apoptosis), leading to the elimination of the deteriorated cells and providing stability to the genome [4]. Fibroblasts extracted from radiosensitive patients with AT, Fanconi anemia or other diseases, in cell culture upon irradiation show accelerated telomere shortening (dose range 1–7 Gy) [5] and a high level of replicative aging (~2.5 Gy) [6].

Gene expression analysis is one of the most comprehensive approaches to determine a primary response of a cell to stress. However, up to now there have been relatively few studies aiming to assess the cell response to IR at the transcription level [2,7–9]. One such article [10] describes the gene expression analysis of fibroblasts AG01522, irradiated with doses 5 cGy and 2 Gy.

In our previous study we focused on the aging processes of the fibroblasts [11], we analysed the transcriptome of cells extracted from young, middle-aged and old subjects as well as subjects with progeria, in order to find similarities between the signalomes of old people and of progeroid patients, as well as with replicatively aged cells.

Here we compare the transcriptomes of fibroblasts irradiated with different doses of IR (Marthandan et al. 2016) with replicatively aged fibroblasts and those taken from different patients, as in [11]. Our data demonstrate that the transcriptome of replicatively aged fibroblasts is more similar to the transcriptome of the cells irradiated with 2 Gy, than with 5 сGy.

## RESULTS

### Effect of low and high doses of radiation on cells: analysis of differentially expressed genes and pathways

Gene expression samples from replicatively aged fibroblasts [11] and those taken from different patients [12] were stratified into groups by the number of passages and donor’s age, respectively (Table 1). Samples of fibroblasts that were irradiated with X-ray [9] were stratified into functional groups by the dose and time after exposure (Table 1). For each of the functional groups we discovered significantly differentially expressed genes and signaling pathways, identified by the iPanda algorithm [12] and listed in the Table 1.

**Table 1:** Analysis of differentially expressed genes and metabolic pathways for the selected datasets.

**Study**	**Age groups**	**Irradiation dose, time after exposure**	**Differentially expressed genes,** ***p-value* < 0.01**	**Signaling pathways,** ***p-value* < 0.05**
EMTAB2086_70_vs_30	70/30 cycles	-	1676	387
EMTAB2086_80_vs_30	80/30 cycles	-	4217	1328
GSE55118_middle_vs_young	30-50/<30 years	-	603	29
GSE55118_old_vs_young	>50/<30 years	-	442	24
GSE59861_12h_2Gy	-	12 h., 2 Gy	485	24
GSE59861_12h_5cGy	-	12 h., 5 cGy	359	11
GSE59861_24h_2Gy	-	24 h., 2 Gy	1201	108
GSE59861_24h_5cGy	-	24 h., 5 cGy	905	41
GSE59861_3h_2Gy	-	3 h., 2 Gy	555	44
GSE59861_3h_5cGy	-	3 h., 5 cGy	70	0
GSE59861_6h_2Gy	-	6 h., 2 Gy	166	2
GSE59861_6h_5cGy	-	6 h., 5 cGy	150	4

**Figure 1:**
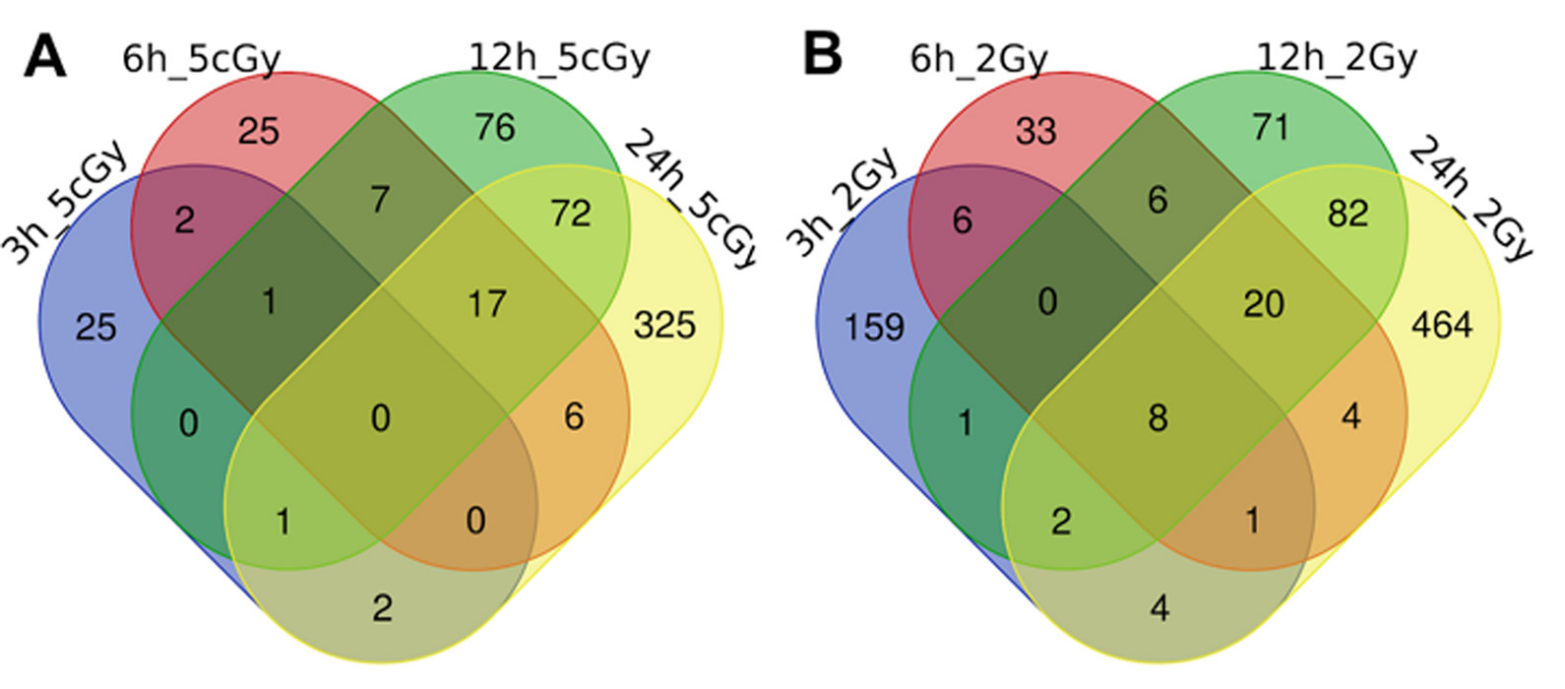
Venn diagrams, illustrating overlapping effects (up-regulated genes) of IR at different times of exposure: 3, 6, 12, 24 hours for the doses 5 cGy (**A**) and 2 Gy (**B**). Numbers indicate the amount of common/unique differentially expressed genes for the studied groups (Table S1).

**Figure 2:**
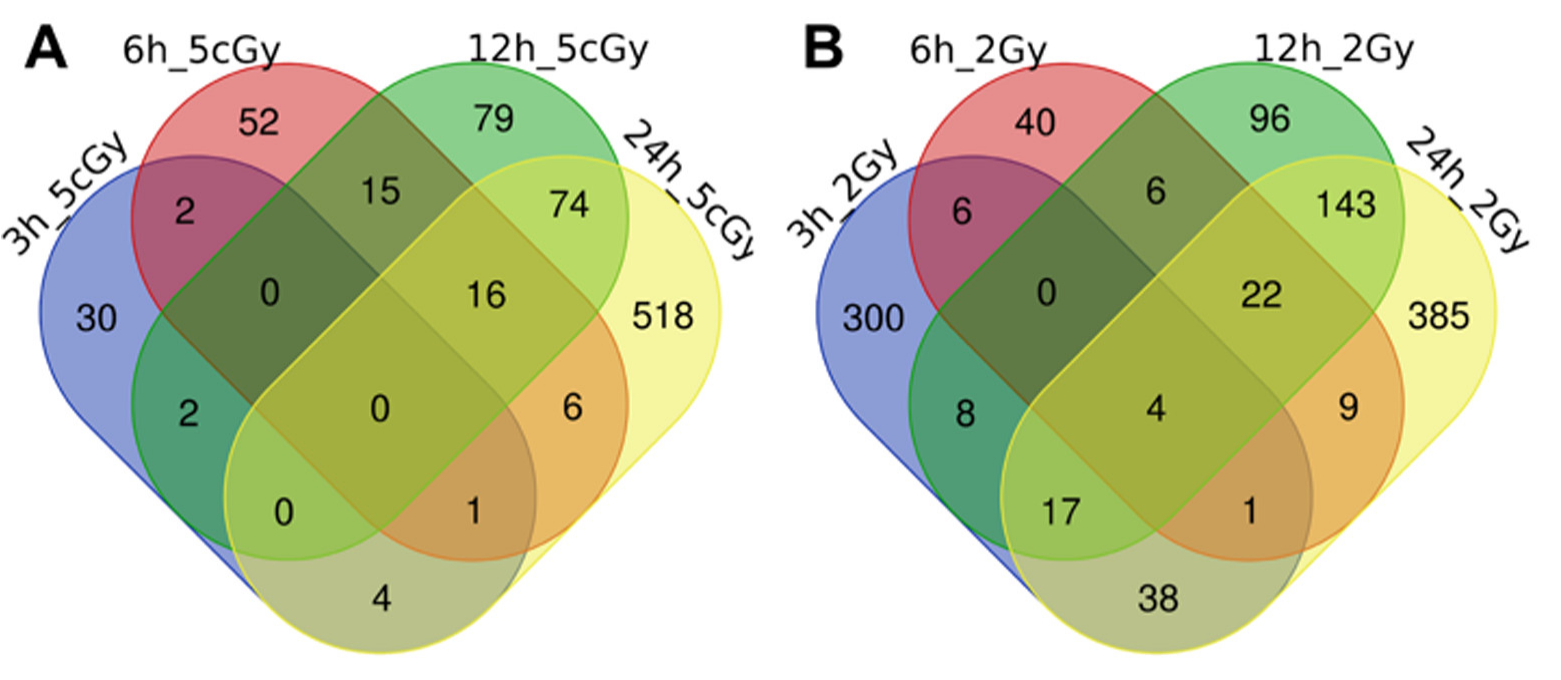
Venn diagrams, illustrating overlapping effects (down-regulated genes) of IR at different times of exposure: 3, 6, 12, 24 hours for the doses 5 cGy (**A**) and 2 Gy (**B**). Numbers indicate the amount of common/unique differentially expressed genes for the studied groups (Table S2).

Here we present the results of the comparison of differentially expressed genes for doses 5 cGy and 2 Gy as a time series, grouped by time after exposure to IR (3, 6, 12 and 24 hours, Figure 1 and Figure 2, Table S1, Table S2).

Exposure of cells to 5 cGy of X-ray 3 hours after irradiation leads to the activation of the genes involved in connective tissue/extracellular matrix remodelling; in particular, we found upregulated genes responsible for lipids biosynthesis: ceramides, sphingolipids and phosphatidylinositol phosphate. Also, among the other up-regulated genes, we found genes responsible for the development of alveoli, as well as the FGF22, which plays a role in the regulation of cell growth and development [13]. Among down-regulated genes were *CEP152*, necessary for centriole assembly [14], and *DYNLRB2*, involved in the synthesis of new organelles and mitotic spindle organization [15].

According to GO analysis, 6 hours after X-ray exposure to 5 cGy, further processes occur in the cells: cell cycle arrest (*IRF6*, *DDIT3*) [16], apoptosis signalling and programmed cell death (*DDIT3*, *DDIT4*, *TRAF1*, *SPATA2*) [17,18], development of the cell response to ROS (SESN2, DDIT4) [19], arrest of the translation in the endoplasmic reticulum in response to cellular stress, DNA damage response (*DDIT4*), etc. At the same time there is a suppression of the expression of genes involved in cytokines productions (*EGR1, BIRC3, HSPA1A, CCL1, LY9*), MAP-kinase cascade (*TAB2, DUSP1, DUSP2, KLHDC10*), protein folding and their transport across the endoplasmic reticulum.

Signalling pathways activation analysis showed that proteasomal protein degradation and EGF-Rab5 (linked to endosomes internalisation and extracellular signalling) pathways are up-regulated [20] (Table S3). At the same time, protein folding and cytoskeleton development pathways are down-regulated (Table S4).

12 hours after exposure to 5 cGy of X-ray we still observe high expression level of genes involved in programmed cell death (*DAB2IP, SFRP2, CHAC1, RRAGC, RFFL, DDIT4, OGT, C4ORF14, ATF4, KRT36, TRAF1*), while DNA replication genes (*CCNL1, CDC6, SLFN11, MSH6*), ROS-responsive genes (*DUSP1, FOS, PPIF*), and genes allowing G1/S transition (*CCNL1, CDC6, CCND1*) are suppressed. For this time point, analyses demonstrate a strong up-regulation of the Wnt/Notch pathway, linked to apoptosis and the protein degradation processes [21] (Table S3).

Signalling pathways activation analysis showed that, even 24 hours after the exposure, the Wnt/Notch pathway is still hyper-activated. In addition, we observed an increased activation of a number of signaling pathways associated with syndecan (Table S3). Syndecans mediate cell adhesion, cytoskeleton reorganization, and cellular signaling due to their function as co-receptors for many ligands including FGF, VEGF, TGFβ and fibronectin [22]. In particular, syndecans play an important role in extracellular matrix remodelling and wound healing [23,24]. In accordance with that, we also observed up-regulation of pathways linked to the activation of EGFR, VEGFR1 and VEGFR2 receptors, the Hedgehog pathway (important for cell differentiation [25]), as well as pathways involved in the positive regulation of cell adhesion and glucose uptake (Table S3). Taken together, our data suggest that by 24 hours after exposure to 5 cGy of X-ray the processes of cellular degradation and programmed cell death are replaced by cellular regeneration.

Exposure of the fibroblasts to 2 Gy of X-ray 3 hours after irradiation results in the development of the classical irradiation response, up-regulation of *POLH*, *CDKN1A*, *MTA1*, *GADD45A*, *AEN* [26]: simultaneous expression of proteins responsible for cell cycle arrest *CDKN1A*, *GADD45A* and DNA repair, *POLH*. Pathway analysis shows that X-ray exposure induces p53-mediated DNA damage response, BRCA1-mediated cell cycle arrest and activates PI3K/Akt pathway. At the same time, pathways related to syndecans and Catenin Beta 1, and those involved in cell adhesion and proliferation are suppressed [27].

6 hours after exposure to 2 Gy of X-ray we still observe stress-response in the cells. In particular, apoptotic processes are induced: *TRIAP1, PHLDA1, FAS, CDKN1A,TP53INP1,* etc. Analysis shows up-regulation of genes responsible for cellular aging (senescence): *TGFB3, DKK1, GCLC, KCNMB1, CDKN1A, DDC, MYST3* [28–30]. In parallel, cells activate defence mechanisms against ROS and DNA damage: glutathione is synthesized (*CHAC1, GCLC* и др.) [31], polymerase *POLH* and *PCNA* factor expression levels are enhanced.

12 hours after exposure we still observe signs of apoptosis: up-regulated expression of *DAB2IP, SFRP2, ZMAT3, BCL6, TP53INP1, PHLDA3, DDIT4, TOP2A, BBC3, CDKN1A, APH1B, ATF4, SCIN.* In parallel, an irradiation response is unfolding, similar to the one we saw 6 h after exposure: analysis shows activation of *POLH, DDB2,* *TOP2A*, responsible for double-stranded break (DSB) repair. At the same time, we observe a down-regulation of genes important for DNA replication (*CCNE2, MCM10, GINS3, MCM3, etc*.) [32], protein biosynthesis and G1/S transition (*CCND1, CCNE2*, etc.) [33]. Analysis of signalling pathways showed activation of the Wnt/Notch pathway, BRCA1 G1/S cell cycle arrest, p53-mediated DNA damage response, and the degradation of AKAP1 protein, which plays an important role in the regulation of protein kinase A function and its cellular distribution (Table S3) [34]. In particular, it has been shown that AKAP1 is necessary for the maintenance of mitochondrial homeostasis and, as a consequence, for the regulation of oxidative phosphorylation and senescence [35]. At the same time we observe down-regulation of the pre-replicative complex through the inhibition of the CDC6/ORC pathway (Table S4) [36] and the suppression of DNA single-strand breaks (SSB) repair processes.

24 hours after exposure to 2 Gy of X-ray we observe initiation of cell responses to ROS (*SESN1, SESN2*) [19] and cell starvation, leading to lipids breakdown (*AKR1C3*). We observe activation of catabolic processes, autophagy (*TRIM22, TP53INP1*) and suppression of transcription and translation. At the level of the signalling pathways, we observe strong (equal to the 12-hour level) deprivation of DNA replication, DNA SSB repair, and mitosis (G1/S transition, mitotic spindle formation, cyclins synthesis). On the other hand, pathways related to apoptosis (Notch/Wnt, ATM) and mitotic arrest are up-regulated (Table S3).

Comparison of gene expression of the fibroblasts exposed to different X-ray doses at the same time point after irradiation (Table S1 and Table S2) shows that: 3 hours after exposure there are just 11 commonly expressed genes (4 up- and 7 down-regulated), which complicates proper analysis of the common pathways; 6 hours after exposure there are 5 commonly up-regulated genes and no common down-regulated genes; 12 hours after irradiation for both doses we observe suppression of replication and transcription (*KLF4, VEGFA, ZNF691, DAB2IP*), and enhancement of p38-MAPK, Wnt and VEGF pathway activities (Table S3); 24 hours after exposure to X-ray, among common processes, we detect stimulation of hydrogen peroxide decomposition and glutathione synthesis (protection against ROS), while genes involved in DNA replication and G/S transition are suppressed, as well as gene expression in general.

### Comparison of ageing with the effect of low and high doses of radiation on the cells

**Figure 3:**
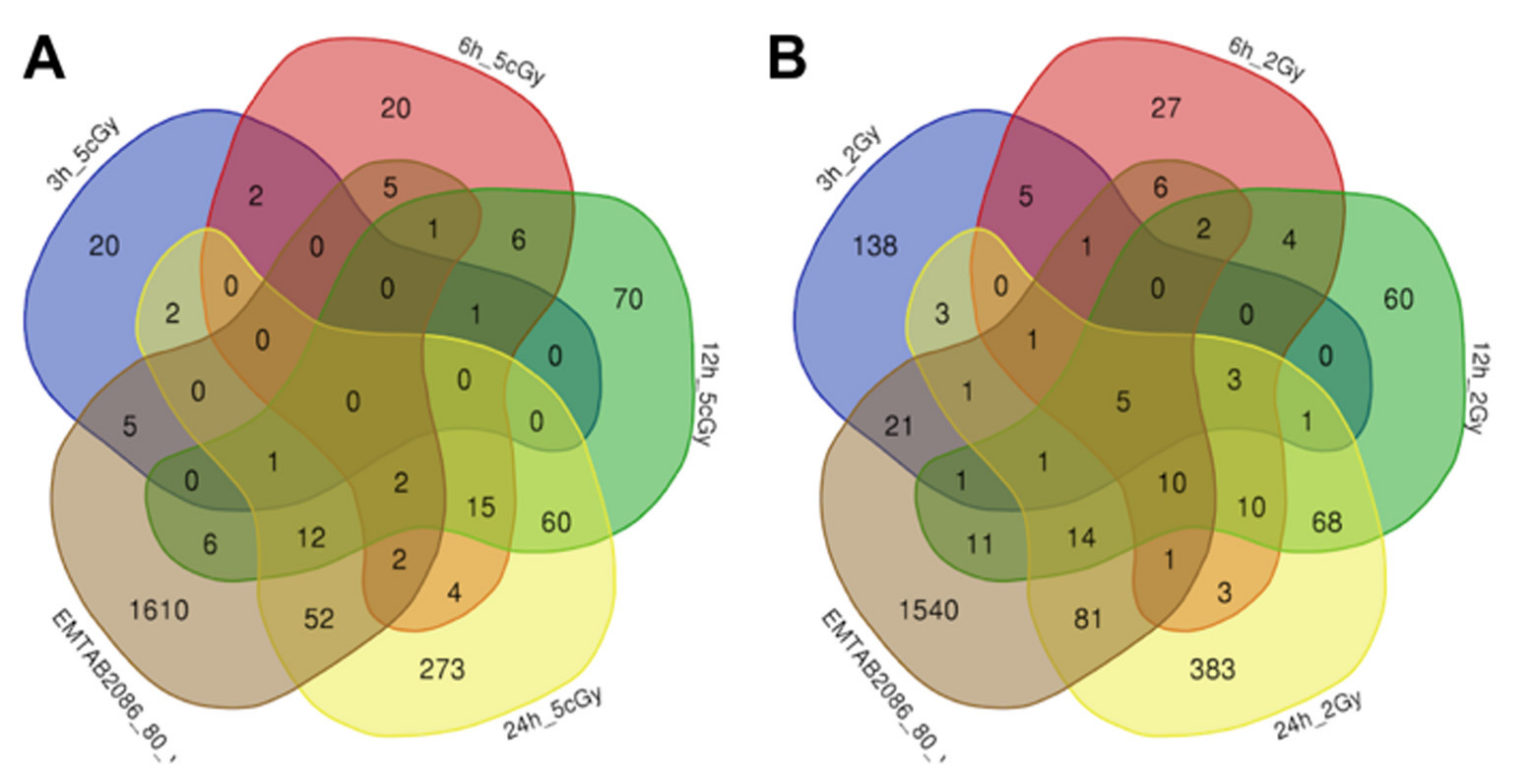
Venn diagrams, illustrating overlapping effects (up-regulated genes) of replicative aging and IR at different times of exposure: 3, 6, 12, 24 hours for the doses 5 cGy (**A**) and 2 Gy (**B**). Numbers indicate the amount of common/unique differentially expressed genes for the studied groups (Table S1).

**Figure 4:**
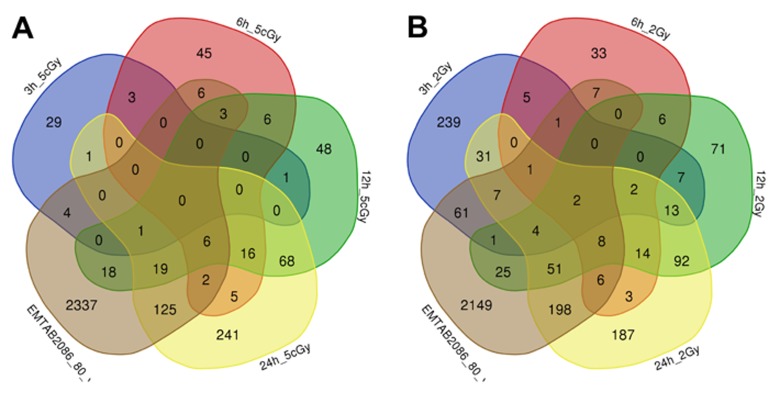
Venn diagrams, illustrating overlapping effects (down-regulated genes) of replicative aging and IR at different times of exposure: 3, 6, 12, 24 hours for the doses 5 cGy (**A**) and 2 Gy (**B**). Numbers indicate the amount of common/unique differentially expressed genes for the studied groups (Table S2).

Comparison of the data on gene expression in fibroblasts 3 hours after exposure to 2 Gy X-ray with replicatively aged cells (data sets EMTAB2086_70_vs_30 and EMTAB2086_80_vs_30) shows that, among the common differentially expressed genes, there is an up-regulation of genes involved in DNA damage signalling through p53 pathway (*BTG2, SESN2, CDKN1A, GADD45A*), as well as the general stress response (*EPHA2, BTG2, SESN2, SESN1, CDKN1A, EDEM3, GADD45A*). Among commonly down-regulated ones, we found genes responsible for cell cycle progression (*CNOT6, CDKN1B, PRKDC, MSH6*); whereas gene *TAOK2*, involved in stress-response [37,38], is in fact up-regulated Table S2, Table S3, Figures 3, 4).

Comparison of gene expression 6 hours after exposure to 2 Gy X-ray with replicatively aged cells show that both of them slow down cellular metabolism because of the lack of amino acids, so-called cellular starvation (*SESN2, SESN1, FAS, CDKN1A*), along with an initiation of apoptosis in response to external/internal signals (*MDM2, BCL2L1, TP53INP1, CDKN1A, TNFRSF10B, FAS, BLOC1S2*) and DNA damage signals. At this time point, down-regulated genes for 2 Gy are those involved in cleavage of intra-strand DNA crosslinks and DNA repair (*RFWD3, ERCC3, HMGB1, FANCE, FANCL, MSH6*); down-regulated genes for 5 cGy are those involved in cell proliferation (*EGR1, TXNRD1, TCF7L2, MRPS27, HSPD1, EPS8*).

Results of comparison of gene expression 12 hours after irradiation (2 Gy) with replicatively aged fibroblasts demonstrates suppression of cell p53-mediated DNA damage response; among common up-regulated we found genes involved in apoptosis and response to radiation (*MDM2, BCL2L1, TP53INP1, CDKN1A*). Common differentially expressed genes for replicatively aged cells and fibroblasts irradiated with 5 cGy are signals about external stimuli (*EGR2, SESN2, GDF15*). Down-regulated genes for 2 Gy are those involved in DNA replication (20 common genes), DNA repair and initiation of mitosis (7 and 13 common genes). Suppressed genes for 5 cGy are cell cycle genes.

Common differentially expressed genes for fibroblasts 24 hours after irradiation (5 cGy and 2 Gy doses) and fibroblasts from old patients, as well as replicatively aged cells are presented in Table S3 and Table S4. Comparison of gene expression 24 hours after irradiation (2 Gy) with data sets EMTAB2086_70_vs_30 and EMTAB2086_80_vs_30 reveals that there are some shared up-regulated genes: genes involved in cell death (17 genes), p53-mediated DNA damage response (9 genes), as well as genes involved in cell cycle arrest (5 genes) and autophagy (6 genes). Common downregulated genes (2 Gy) are those which play a role in mitosis (including replication and formation of mitotic spindle) and DNA repair. For smaller dosed (5 cGy) and replicatively aged cells, common responses include MAP-kinase signalling (4 genes), activation of other signalling cascades (5 genes), down-regulation of genes involved in DNA replication (over 30 genes), cell division (15 genes) and DNA DSB repair.

Signaling pathway analysis revealed the most relevant results for the differential expression/activation of signalling pathways in the cells 24 hours after exposure to IR (Table S3, Table S4). The results of the comparison of gene expression in replicatively old fibroblasts (data sets EMTAB2086_70_vs_30 and EMTAB2086_80_vs_30) and in cells exposed to 5 cGy of X-ray (24 hours after irradiation) demonstrate several common trends for both data sets, particularly an activation of signalling pathways important for cell cycle and adsorption of salts/ions, while comparison to the effects of 2 Gy 24 hours after exposure shows, among common activated pathways, G1/S cell cycle arrest and DNA damage response (BRCA1/E2 pathway).

We have not found any common differentially expressed genes in irradiated fibroblasts and fibroblasts obtained from middle-aged patients (GSE55118_middle_ vs_young), however there were some common changes when we compared them to “old” fibroblasts (GSE55118_old_vs_young). In particular, for the X-ray dose of 2 Gy we observe common down-regulation of pathways involved in cell division: chromosome segregation, centrosomes separation in mitosis, mitotic spindle formation, as well as GATA2-mediated transcription (Table S4). Among shared up-regulated pathways, when we compared “old” fibroblasts to those irradiated by the X-ray dose of 5 cGy, we observe the above-mentioned cell adhesion pathways, while the GATA2 pathway is down-regulated

## DISCUSSION

The above results of the comparison of irradiated fibroblasts, replicatively aged fibroblasts and fibroblasts from old patients allow us to understand not only the cellular response to low and high doses of X-ray, but also the dynamics of this process.

Irradiation of cells with 5 cGy of X-ray induces detectable cellular response just 6 hours after exposure: it causes up-regulation of genes involved in the cell cycle arrest, down-regulation of DNA replication and repair, and a response to cellular ROS. Analysis of signalling pathways 24 hours after irradiation shows up-regulation of the Wnt/Notch pathway, involved in apoptosis. However, along with the apoptotic response, we show up-regulation of several signaling pathways related to syndecans, which are important for ECM remodelling and wound healing, and stimulation of signaling from EGFR, VEGFR1 and VEGFR2, up-regulation of Hedgehog-pathway, cell adhesion and glucose uptake. Taken together, these data suggest that 24 hours after exposure to 5 cGy, cell degradation and apoptosis processes progressively switch to cell regeneration.

X-ray exposure of fibroblasts to 2 Gy causes changes at the transcriptional level as early as 3 hours after irradiation: genes involved in DNA DSB and SSB repair and cell cycle arrest, are upregulated. Analysis of signaling pathways demonstrates activation of the p53-mediated DNA damage response, the BRCA1-mediated cell-cycle response, and of the PI3K/Akt pathway. 6 hours after exposure to IR we observe, in addition to above-mentioned, the activation of genes involved in response to ROS and senescence (aging). 12 hours after irradiation protein biosynthesis is shutting down, and 24 hours after exposure cells initiate response to cell starvation, which leads to lipids breakdown, catabolic processes and autophagy, down-regulation of transcription and translation and sensitization of pathways involved in apoptosis (Notch/Wnt, ATM) and cell cycle arrest. Thus, we can conclude, that 24 hours after exposure to 2 Gy of X-ray, cells die, and do not regenerate.

Overall, on the level of gene expression and signaling pathways, the replicatively aged cells are more similar to fibroblasts exposed to 2 Gy of X-ray, than to cells irradiated with 5 cGy. 3 hours after exposure to 2 Gy of X-rays (unlike 5 cGy), the irradiated fibroblasts acquire some characteristics of replicatively aged cells, with 108 common differentially expressed genes (compared to 10 for 5 cGy); in particular, cells initiate p53-mediated DNA damage response, stress-response, as well as cell cycle arrest with a consequent decrease of mitotic activity. 6 and 12 hours after exposure to 2 Gy of X-rays, the fibroblasts have 186 common differentially expressed genes with replicatively aged cells (compared to 96 for those, exposed to 5 cGy). Most of these genes, according to GO analysis, are involved in apoptosis, cell cycle arrest, down-regulation of replication and transcription, as well as the cellular response to radiation, DNA damage, cell starvation and ROS. For the dose of 5 cGy and replicatively aged cells, the only common down-regulated pathway (according to GO) was associated with the cell proliferation. 24 hours after exposure to 2 Gy of X-rays, the gene expression profile of the fibroblasts shows an up-regulation of genes responsible for execution of cell death, p53-mediated DNA damage response, cell cycle arrest and autophagy. At the same time, there are suppressed genes involved in DNA repair and mitosis (including replication, mitotic spindle formation, etc). For the lower dose, 5 cGy, cell response is mostly based on the up-regulation of MAP-kinase signaling and down-regulation of genes important for DNA replication, cell division and DNA DSB repair.

According to our GO analysis [39] (Table S5) and pathway analyses (Table S3, Table S4) 24 hours after the irradiation cells have strong negative regulation of genes associated with cell cycle, DNA repair and replication for low and high doses and positive regulation for genes responsible for programmed cell death and p53-mediated response in case of high dose and signal transduction, cell adhesion, communication. 

Our results are in good agreement with previous study (see Table S5) [40] of fibroblasts undergoing replicative and radiation γ-irradiation induced senescence. Authors state that senescence induced by both factors is connected with cellular response to damage and “cell cycle” pathway was downregulated for both states while replication errors were essential for the induction of replicative senescence but not for irradiation induced senescence [40]. Additionally to these results we determined the differences between the doses of the irradiation and signaling pathways involved in cellular response.

Overall this study provides a comprehensive analysis of signalome changes caused by the processes of aging and ionizing radiation. Signaling pathways identified in this study provide a valuable common mechanistic link between two processes and can be used to develop new generation of gero- and radioprotectors.

## METHODS

### Description of datasets used to assess the detrimental effects of high doses of ionizing radiation

Datasets selected for the analysis of replicative aging of fibroblasts [41] are the same as those described in the previous study [11]: GSE55118, E-MTAB-2086 from Lackner et al. [41].

For the analysis of irradiated fibroblasts we selected dataset GSE59861 [40], that consists of gene expression data for 1522 human fibroblasts subjected to two doses of X-ray irradiation: 5 cGy and 2 Gy, and analysed at five time-points: 0 h, 3 h, 6 h, 12 h, 24 h.

Each pre-processed gene expression dataset was analyzed independently of the others, using the iPanda algorithm [12]. We selected further groups for the analysis:

1) replicative aging 70 cycles versus 30 cycles and 80 versus 30: EMTAB2086_70_vs_30 and EMTAB2086_80_vs_30 respectively

2) “middle-aged” and “young” fibroblasts GSE55118_middle_vs_young

3) “old” and “young” fibroblasts GSE55118_old_vs_young

4) irradiated fibroblasts GSE59861_3h (6h,12h,24h)_2Gy(5cGy)

For the studied groups, differentially expressed genes and dysregulated signaling pathways were identified, as well as activated pathways, as described previously in [11]. Only genes present in all the samples were taken into account for analysis.

## Supplementary Material

Supplemental Table S1

Supplemental Table S2

Supplemental Table S3

Supplemental Table S4

Supplemental Table S5
